# A Longitudinal Examination of Stress, Affect Dynamics, and Alcohol-Related Outcomes Across Emerging Adulthood

**DOI:** 10.3390/bs15080998

**Published:** 2025-07-22

**Authors:** Stephen Armeli, Richard Feinn, Elise Bragard, Howard Tennen

**Affiliations:** 1 School of Psychology and Counseling, Fairleigh Dickinson University, Teaneck, NJ 07666, USA; armeli@fdu.edu; 2 Department of Medical Sciences, Quinnipiac University, Hamden, CT 06518, USA; richard.feinn@quinnipiac.edu; 3 Alcohol Research Center, University of Connecticut School of Medicine, Farmington, CT 06030, USA; bragard@uchc.edu; 4 Department of Public Health Sciences, University of Connecticut School of Medicine, Farmington, CT 06030, USA

**Keywords:** alcohol, drinking motives, affect dynamics, longitudinal

## Abstract

We examined the associations between individual differences in intensive longitudinal data-derived affective dynamics (i.e., positive and negative affect variability and inertia and positive affect–negative affect bipolarity) and concurrent stress, drinking levels, and affect-regulation drinking motives across three time points spanning early adulthood. This allowed us to evaluate the stability of the affective dynamics and whether their associations with alcohol outcomes varied across this critical developmental period. Moderate-to-heavy college drinkers (N = 1139, 51% women) reported on their affective states, stress, drinking levels, and drinking motives daily for 30 days using a web-based daily diary in three assessment waves: during college and at two post-college waves, approximately 5 and 10 years after the initial assessment. Findings indicated moderate stability of the affect dynamic indicators, except for inertia. Negative affect variability showed the strongest positive association with mean daily stress. Individuals who demonstrated stronger affect bipolarity had lower drinking levels and higher enhancement motivation. None of the other dynamic indicators were consistently related to the drinking outcomes in the predicted direction after controlling for mean affect levels, and we found little evidence for changes in these effects across time. Our results add to the inconsistent literature regarding the associations between affective dynamics and alcohol-related outcomes.

## 1. Introduction

Life stress and negative affect have long been thought to be risk factors for problematic alcohol use (Greeley & Oei, 1999), though evidence for meaningful associations between these variables is mixed at best (Tovmasyan et al., 2022a, 2022b). The challenges in demonstrating links between these variables have been clearly highlighted over the last several decades, during which researchers have turned to intensive longitudinal research designs (Tennen et al., 2000; Iida et al., 2023) in an attempt to model in everyday life the ebb and flow of daily and within-day alcohol use as a function of changes in stress and affect levels. This line of research has produced equivocal findings. Indeed, Dora et al.’s (2023) recent meta-analysis of data from intensive longitudinal studies showed no meaningful association between daily changes in negative affect and concurrent drinking levels.

Other researchers have attempted to explain variation in drinking-related outcomes by leveraging data from intensive longitudinal designs to model more dynamic affect-related processes indicative of emotion dysregulation. For example, dysregulated affect is not only indicated by high levels of negative affect and low levels of positive affect, but also by high levels of affect variability (e.g., Eid & Diener, 1999), greater negative affect inertia or resistance to change (e.g., Koval et al., 2013), and the degree to which positive and negative affect are correlated within a person, i.e., bipolarity, with stronger inverse associations being indicative of dysregulation (e.g., Dejonckheere et al., 2018; Houben et al., 2015). These general types of affect dynamics are consistent with core factors identified via factor analysis of 14 commonly examined intensive longitudinal data-derived (ILDD) affect dynamics indicators in Dejonckheere et al.’s (2019) meta-analysis.

With respect to alcohol use and related outcomes, individual differences in ILDD affect variability are the most commonly examined, though findings from this line of research have been mixed. Several studies have found that individuals who show greater levels of daily or weekly affect variability report higher drinking levels (Gottfredson & Hussong, 2013; Linn et al., 2024; Rankin & Maggs, 2006; Mohr et al., 2015; Rahal et al., 2024; Weiss et al., 2018; cf., Peacock et al., 2015). Gottfredson and Hussong (2013) also found that affect variability was positively associated with drinking-to-cope (DTC) motivation, which is consistent with the notion that such indicators do indeed reflect aspects of dysregulation for which individuals use alcohol as a form of self-medication. However, few studies (cf., Gottfredson & Hussong, 2013; Rahal et al., 2024) found these associations after controlling for mean affect levels, which are often highly correlated with affect variability and have been shown to confound associations between affective dynamics and outcomes such as depressive symptoms (Dejonckheere et al., 2019) and Big Five personality characteristics (e.g., Wendt et al., 2020). Dejonckheere et al. (2019) argue that, given mean affect levels are foundational and highly predictive of well-being outcomes, failing to control for them when evaluating the effects of dynamic affect indicators renders the unique contributions of such predictors ambiguous.

To date, Feinn et al. (2023) conducted the most comprehensive examination of the associations between individual differences in a variety of ILDD affective dynamics and alcohol-related outcomes. In their study of a large sample (*N* = 1640) of college student drinkers, they examined whether drinking level and affect-regulation drinking motives—i.e., DTC with stress and negative affect and drinking to enhance positive emotions—were related to affect variability, affect inertia, and affect bipolarity derived from 30 days of daily reporting. The results indicated that after controlling for mean levels of positive and negative affect, none of the dynamic indicators uniquely predicted the drinking-related outcomes except for affect bipolarity. However, this effect was in the opposite direction to prediction for drinking level, with individuals who showed weaker negative associations between positive and negative affect (i.e., less bipolarity) exhibiting higher drinking levels.

In the present study, we attempted to further this line of research by examining a sub-set of individuals from Feinn et al. (2023) who subsequently participated in two follow-up assessment waves. Specifically, moderate-to-heavy drinkers during college were assessed using an identical daily reporting protocol after exiting the college environment, approximately five years after the initial assessment, and a third time in their late 20s/early 30s, approximately five years after the post-college assessment. This longitudinal burst design allowed us to address several shortcomings of Feinn et al. (2023) specifically, and of the broader literature examining ILDD affective dynamics more generally. First, Feinn et al. (2023) suggested that the null effects for the affect dynamics obtained during college years might be related to the fact that college student drinking tends to be more closely linked to social and normative factors (Gonzalez, 2012; Mohr et al., 2001) rather than negative reinforcement processes. Consistent with research showing that coping-related drinking becomes more closely related to problematic alcohol use as individuals progress through early adulthood (e.g., Littlefield et al., 2010), it is possible that individual differences in affect dysregulation, as indicated by these ILDD indicators, would show stronger associations with drinking-related outcomes in the post-college assessment waves.

Second, our longitudinal burst design allowed us to further examine the psychometric properties of our indicators of affective dynamics. Indeed, the unreliability of these indicators might have attenuated any observed effects of interest in Feinn et al. (2023). To date, relatively little research has examined the stability of ILDD dynamic affect indicators. Although some studies provide evidence of significant trait variability in such indicators (e.g., Alessandri et al., 2021; Eid & Diener, 1999; Larson, 1983; Penner et al., 1994), few studies have examined their stability beyond a few weeks, and those that have, show some evidence for rank-order stability. For example, Dejonckheere et al. (2018) found moderate-sized stability coefficients (*r*s in the 0.30–0.40 range) for ILDD bipolarity assessed at six months and one year. Alessandri et al. (2021) found a moderate association (*r* = 0.28) between ILDD negative affect inertia calculated across two daily reporting bursts separated by a month. In one of the longest longitudinal studies, Hardy and Segerstrom (2017) found a moderate-sized association (*r* = 0.37) for negative affect variability and a weak to moderate association (*r* = 0.19) for positive affect variability across ten years. In our longitudinal burst design, we estimated ILDD indicators of affect variability, inertia, and bipolarity for the same individuals over three distal time points each separated by five years.

Finally, we also examined whether the dynamic indicators were related to concurrent life stress assessed via daily reporting. Demonstrating that individuals with greater affect variability, inertia, and bipolarity had higher average levels of stress during the daily sampling periods used to derive the dynamic indicators would provide evidence for their construct validity.

## 2. Method

### 2.1. Participants and Procedure

College students were initially recruited from a large eastern state university for a study on alcohol use and daily experiences. For the larger study, individuals needed to have consumed alcohol at least twice in the past month and not have received treatment for alcohol use. Moderate-to-heavy drinkers from the initial sample—i.e., individuals who reported at least one heavy drinking day (≥4 drinks for women and ≥5 drinks for men) in both a 30-day retrospective assessment and a 30-day daily diary reporting phase (each assessment covering two different time points in college (*N* = 1141)—were then recruited for two follow-up assessment waves. At wave 2, approximately five years after the initial assessment, participants needed to have graduated or no longer be working toward an undergraduate degree. Wave 3 occurred approximately five years after wave 2. Participants were reimbursed for their time completing the baseline and daily surveys: up to USD 135 at wave 1, USD 210 at wave 2, and USD 265 at wave 3. The institutional review board approved all procedures.

At all waves, participants first logged on to a secure website, where they provided informed consent and completed a baseline survey including demographic information. Approximately two weeks later, participants completed the 30-day daily diary portion of the study, in which they reported each day on their alcohol use, drinking motives, perceived stress, and affect. Daily surveys were completed each day between 2:30 p.m. and 7:00 p.m. in wave 1 and between 4:00 p.m. and 8:30 p.m. in waves 2 and 3. These reporting windows were selected to coincide with most participants’ naturally occurring end of school/workday but before typical evening activities begin (including drinking). We included all participants in our final analyses regardless of the number of waves completed, as long as they met our minimum daily reporting adherence criteria of 15 days (for each wave). Specifically, we had 1139 individuals available from wave 1, 906 from wave 2, and 797 from wave 3; 65.1% had data from 3 waves and 84.5% had data from at least 2 waves. Daily diary adherence was good, with the mean number of daily reports completed being 26.0 days (*SD* = 4.0) in wave 1, 27.9 days (*SD* = 3.4) in wave 2, and 29.2 days (*SD* = 2.5) in wave 3.

At wave 1, the sample was 51.3% women and predominantly White (84.8%); the mean age was 19.2 years (*SD* = 1.4) and most (73.0%) were in their freshmen or sophomore years of college. At wave 2, the mean age was 24.6 years (*SD* = 1.3). The majority were employed full-time (78.5%) and had at least a bachelor’s degree (97.6%). Only 4.6% were married and 1.1% had children. At wave 3, the mean age was 31.0 years (*SD* = 1.3). The majority were employed full-time (95.4%), 49.2% were married, and 23.7% had children.

### 2.2. Measures

*Daily drinking and motives*. On each of the 30 daily diary days, participants logged in to a secure Internet-based daily survey to report how many drinks (responses: 0 to >15) they had in social (interacting with others) and non-social (alone; not interacting with others) contexts separately for the previous evening (i.e., after they completed the prior day’s survey) and for the current day (up to the reporting time). One drink was listed as “*one 12-oz. can or bottle of beer, one 4-oz. glass of wine, one 12-oz. wine cooler or 1-oz. of liquor straight or in a mixed drink*.” We summed the social and non-social drinks for each period, then calculated daytime and nighttime drinking values to create a total daily drinks variable. To avoid extreme values from exerting undue influences, we recoded days on which more than 15 drinks were reported to a maximum of 15. We then averaged daily values across all available reporting days to create a mean daily drinking variable.

On days when participants reported alcohol use for either the previous night or the current day (which occurred in separate sections of the daily survey), they were then asked about their reasons for drinking during those time periods using a slightly modified version of Cooper’s (1994) Reasons for Drinking scale. Participants were asked whether they drank for the following reasons (responding with a 3-point scale [0 = *no*, 1 = *somewhat*, 2 = *definitely*]). DTC motivation was assessed with the items “*to forget my ongoing problems/worries*,” “*to feel less depressed*,” “*to feel less nervous*,” “*to avoid dealing with my ongoing problems,*” “*to cheer up*,” and “*to feel more confident/sure of myself*”. The main alteration was that in the original scale, drinking to reduce anxiety and depression is assessed with a single item; in the present study, this was separated into two items for aims unrelated to the present study. Enhancement motivation was assessed with the items “*because I like the pleasant feeling*” and “*to have fun*.” Composite scores were created across all day and night drinking occasions by averaging together the relevant items. We then calculated overall mean levels across all daily reports. Reliability estimates for these person-level values were derived from intercept-only models (calculated using HLM software (v6); Raudenbush et al., 2004) separately for each wave. Estimates were as follows: wave 1 (DTC = 0.84, enhancement = 0.76), wave 2 (DTC = 0.86, enhancement = 0.85), and wave 3 (DTC = 0.85, enhancement = 0.87).

*Daily affect*. We assessed daily affect using items derived from the Positive and Negative Affect Schedule-Expanded (PANAS-X; Watson et al., 1988) and Larsen and Diener’s (1992) mood circumplex. Participants were asked to rate how well each of the following words describe how they felt that day, from the time they woke up until the time of reporting. They responded using a 5-point scale (1 = “*not at all*” to 5 = “*extremely*”]). Daily negative affect was assessed with the items *nervous, dejected, irritable, hostile, sad, angry, anxious,* and *tense.* Positive affect was assessed with the items *cheerful, happy, excited, relaxed, enthusiastic, content, calm,* and *energetic*. Overall negative affect (NA) and positive affect (PA) scale scores were created by averaging together the relevant items. The day-level internal consistency (alpha) across waves 1, 2, and 3 was 0.91, 0.92, and 0.92 for the PA scale and 0.86, 0.86, and 0.87 for the NA scale.

*Daily stress*. We assessed daily stress by having participants rate the “*overall stressfulness*” for the previous night (after the previous day’s survey) and for the current day (up to reporting time). Responses were made on a 7-point scale (1 = “*not at all stressful*” to 7 “*extremely stressful*”). We computed an overall mean stress level by averaging together all daily reports.

### 2.3. Analytic Strategy

We used dynamic structural equation modeling (DSEM) in Mplus v8.11 (Asparouhov et al., 2018) to estimate the dynamic affect indicators of interest. Specifically, we estimated the following person-level indicators from the daily data at each wave of our indicators of interest: affect variability was the log transformed variance of PA and NA measures; affect inertia was the autoregressive effect of the PA and NA measures using a one-day lag; and bipolarity was the within-person covariance between PA and NA for each person. We used a two-level random effects model with the Bayes estimator. The Bayes estimator used default noninformative priors, with a PSR convergence of 1.05, MCMC Gibbs sampling, two chains, 50,000 maximum iterations, and median point estimates. Convergence and stationarity were verified with autocorrelation and trace plots. Statistical significance was assessed using one-tailed *p*-values with a 0.025 cutoff from the posterior parameter distributions.

We first estimated bivariate associations among the dynamic affect indicators across waves and with mean levels of stress and affect. Next, we estimated multiple predictor models separately for each outcome (drinks, coping motives, enhancement motives). If participants reported no drinking for a wave, they were excluded from the drinking motive models given they did not report on motives. This resulted in 1.9% of the sample being excluded for the wave 2 motive analyses and 7.6% from wave 3 motive analyses. The parameters for the models across all waves were calculated by using information from individuals from all waves regardless of the number of waves they completed, as Bayesian analysis incorporates missingness into the modelling process (Asparouhov & Muthén, 2010). For each outcome, two models were estimated. We first examined the five dynamic predictors together to assess their association with each of the outcomes at each wave. We then estimated the models that included the dynamic predictors with the addition of mean levels of PA and NA.

Finally, we examined whether the partial slopes from the final models varied across waves. First, we estimated a Wald test contrasting a model with the slope parameters constrained to be equal across waves versus allowing them to vary. A significant effect was followed by pairwise comparisons between the three waves.

## 3. Results

Table 1 shows the descriptive statistics for the study variable across the three assessment waves, and Table 2 shows the stability of the dynamic indicators across the waves. The values for the dynamic affect indicators and mean affect levels correspond to the saved model parameters from the DSEM; the mean stress, drinking, and motive levels were derived from the observed data. All dynamic indicators except NA and PA inertia showed some evidence of temporal stability, with the variability indicators being the most stable, showing moderate associations across time. The mean levels of NA and PA showed moderate-to-strong stability across the waves.

Table 2 also shows correlations between the dynamic indicators and mean daily stress and affect levels at each wave. The mean levels of NA and stress showed strong positive correlations with NA affect variability and weak-to-moderate positive correlations with NA inertia. Bipolarity showed generally weak negative associations with mean daily stress, indicating that higher levels of stress were associated with stronger inverse PA-NA associations (i.e., greater bipolarity). In contrast, bipolarity was generally unrelated to mean levels of NA and showed weak negative associations with mean levels of PA. PA inertia and variability were, for the most part, unrelated or weakly related to mean stress and affect levels. Table 2 also shows mean NA levels had strong positive correlations with mean daily stress, whereas mean PA levels showed weak-to-moderate negative associations with mean daily stress. Correlations among the dynamic predictors at each wave are shown in the *supplementary materials*; associations were generally weak, with the strongest associations being between the PA and NA variance indicators, with correlations ranging from 0.27 to 0.42.

Table 3 shows the results from the models examining the effects of the dynamic indictors (without and with control for mean affect levels) on the alcohol outcomes of interest.

The mean affect levels were the most consistent significant predictors, with NA positively related to DTC motives and PA positively related to enhancement motives across all waves. The mean levels of PA were negatively related to DTC motivation across waves 1 and 2, but not at wave 3.

Across all waves, higher bipolarity was related to higher enhancement motivation and lower drinking levels (except at wave 2). We found few other predicted effects for the dynamic indicators after controlling for mean affect levels (using a one-tailed 0.025 alpha level). For example, negative affect variability was uniquely predictive of DTC motivation in waves 2 and 3, but the direction of this effect changed from positive to negative after controlling for mean affect levels. The only other significant effect found in the final models was at wave 3, with individuals who displayed more PA variability having higher enhancement motivation.

Finally, the tests for changes across waves in the final model coefficients revealed only a significant effect for bipolarity (Wald χ2 [2] = 15.82, *p* < 0.001). Follow-up pairwise comparisons showed that the effect of bipolarity decreased from wave 1 to wave 2 (*p* = 0.007) and increased from wave 2 to wave 3 (*p* < 0.001).

## 4. Discussion

We found little support for our hypotheses that individuals who displayed higher levels of affect variability and inertia would show higher levels of alcohol use and stronger affect-regulation drinking motives after controlling for individual differences in mean affect levels. These effects were generally consistent across three assessment waves spanning college years, immediate post-college life, and towards the end of their early adulthood. We did, however, extend Feinn et al.’s (2023) previous findings, showing that greater affect bipolarity is associated with lower drinking levels but higher enhancement motivation.

One goal of the present study was to examine whether previous findings showing generally null effects of affect dynamics on alcohol-related outcomes using our larger college wave sample (Feinn et al., 2023) might be due to the poor reliability and validity of our dynamic indicators. Specifically, it was unclear whether our indicators, derived from once-daily reporting, assessed stable individual difference factors indicative of emotion dysregulation. Our findings regarding these psychometric issues were mixed. We did find that affect variability and bipolarity indicators were somewhat stable over time, suggesting some trait-like qualities. In addition, mean daily stress levels showed weak-to-moderate associations in the predicted direction with negative affect inertia and bipolarity and strong positive associations with negative affect variability. These findings are consistent with the notion that our dynamic indicators of interest reflect problematic affect regulation, i.e., they are heightened during periods of increased stress. However, we should note that the association between stress levels and negative affect variability could be artifactual, given the strong associations between both variables and negative affect levels. The inertia indicators, in contrast, showed little evidence of temporal stability. However, negative affect inertia was positively associated with concurrent stress levels, as predicted. One possibility is that our inertia measures reflect more state-like rather than trait-like processes, varying as a function of contextual factors.

We also replicated and extended the bipolarity findings reported in Feinn et al. (2023). First, we replicated Feinn et al.’s findings in our study focusing only on the moderate-to-heavy drinkers during college. We also found that these associations held across the follow-up waves, with the exception of the drinking level model in wave 2, although this effect was in the predicted direction. Specifically, individuals who showed greater bipolarity (i.e., stronger inverse PA-NA associations) drank less but showed higher levels of enhancement motivation. In line with Feinn et al.’s interpretation, this could represent distinct pathways through which this type of affect dysregulation might affect drinking behavior. More specifically, deficiencies conveyed by higher bipolarity might be linked to problematic interpersonal functioning, and ultimately to self-selection out of normative social scenarios in which drinking commonly occurs (e.g., parties, bars), thus resulting in lower drinking levels. On the other hand, when high bipolarity individuals drink, they might be more likely to use alcohol as a method for regulating affective states—namely upregulating positive affective states. We also found that the strength of this effect varied across waves, decreasing from college to immediate post-college years, and then increasing from that point to the later post-college years. Given the unpredicted nature of this change pattern, replication is needed. Future studies are needed to test the posited links between bipolarity and interpersonal functioning, and whether among high bipolarity individuals, engagement in enhancement motivated drinking results in greater changes in affective states.

In contrast to the bipolarity results, we found little evidence for the incremental validity of our variability and inertia indicators with respect to our alcohol outcomes after controlling for mean affect levels. In addition, we found little support for the notion that higher levels of variability and inertia would show stronger positive associations with our alcohol outcomes as individuals progressed through the critical maturing-out period. The only support for the latter prediction was that across waves, positive affect variability was a significant unique predictor of enhancement motivation in the final wave, and we found a similar trend (one-tailed) for negative affect inertia with respect to drinking-to-cope motivation. However, these coefficients did not vary significantly across waves, and this might simply represent spurious effects given the number of tests we conducted. Nevertheless, these patterns are somewhat consistent with the findings (Littlefield et al., 2010) suggesting that affective processes indicative of dysregulation might become more closely linked to maladaptive drinking as individuals progress through this developmental period.

More problematic was our finding showing that after controlling for mean affect levels, negative affect variability was inversely related to drinking-to-cope motivation—opposite to prediction—in both follow-up waves. We hesitate to offer a substantive interpretation of this finding given its unpredicted nature. However, one possible statistical explanation concerns model misspecification, given (a) the strong positive correlation between negative affect mean levels and variability and (b) the ambiguity pertaining to the actual causal processes underlying the association between mean levels and variability. As Wysocki et al. (2022) demonstrate, the inclusion of statistical control variables that operate in ways other than true confounders can seriously bias parameter estimates. For example, if high levels of negative affect variability and drinking-to-cope motivation (i.e., the predictor and outcomes in our models) are actually causes of mean negative affect levels (our control variable), then mean affect levels should be conceptualized as a collider rather than a confounder and including it the predictive models could bias the effects of affect variability, possibly resulting in the null (and negative) effects we observed.

The possibility that our null results are due to model misspecification is consistent with Linn et al.’s (2024) argument against controlling for mean affect levels when modeling the effects of dynamic indicators, such as affect variability, on outcomes such as alcohol use. Specifically, they argue that affect variability more closely matches the emotion dysregulation mechanisms outlined in theoretical frameworks of alcohol use disorders. Indeed, in our models without mean affect levels as a control, we did find some support for the predicted effects of several dynamic indicators. For example, negative affect inertia and variability were positively related to DTC motivation—as predicted—across all waves. One possibility is that the examination of unique effects of these factors using variable centered approaches is not appropriate because it removes critical commonalities among such factors. Perhaps person-centered approaches would be more useful to identify profiles of individuals based on mean affect levels and dynamic affect factors and examine how various alcohol outcomes vary across these groups. More generally, researchers need to better conceptualize the causal nature among affect dynamics and mean affect levels when modeling their effects on outcomes of interest.

Beyond the issues regarding controlling for mean affect levels, and the possibility that individual differences in these dynamic processes are truly unrelated with these alcohol-related outcomes, we offer several other possible explanations for our results. First, it could be that once-per-day reporting of affective states, especially over a relatively brief reporting window, does not adequately model the dynamic nature of the processes of interest (cf., Rahal et al., 2024). Future studies using intensive approaches that sample affective states in closer temporal proximity (e.g., multiple within-day sampling) and over longer periods of time might provide more accurate assessment of individual difference factors. Second, it could be that the associations between these individual difference factors and alcohol outcomes are more complex, i.e., trait-like dynamic processes might serve as vulnerability factors that interact with other key relevant antecedents of these alcohol outcomes. For example, these dynamic indicators might show stronger effects on drinking-to-cope motivation among individuals with stronger positive alcohol outcome expectancies and maladaptive general coping styles (e.g., Cooper et al., 1995). Future studies with large sample sizes are needed to test such interactions, including possible gender differences in these associations.

Several additional limitations of our study merit mentioning. Given our correlational design, we cannot make any inferences about causality. In addition, the makeup of our sample, being a mostly White and college educated (from one university), limits the generalizability of our findings. We also experienced attrition in our follow-up assessment waves. Although our analytic approach utilized values from all waves in estimating model parameters, it is unclear how the missing data from the follow up waves affected the results. Finally, we used a single item to assess daily stress. Although average daily stress levels were highly related to negative affect levels and several indicators of affect dynamics—thus providing evidence for its validity—future research utilizing multi-item stress measures or more objective biomarkers (e.g., elevated heart rate) would further advance this field.

## 5. Conclusions

We believe that our results add to the ongoing literature regarding the utility of deriving such dynamic indicators from intensive longitudinal designs. Our findings provide some support for the stability of affect variability and bipolarity indicators calculated using once-per-day reports. In addition, we provide evidence for the validity of our negative affect dynamic indicators, given their associations with concurrent stress levels. We also replicated previous findings showing that many of these indicators do not explain variation in alcohol outcomes beyond mean affect levels, and we extended these findings by examining these effects across multiple time points in the critical maturing-out development period spanning college to late twenties/early thirties. We hope that this information will guide future research on this topic.

## Figures and Tables

**Table 1:** Descriptive statistics for study variables across waves.

	*Wave 1*		*Wave 2*		*Wave 3*	
*Measure*	*M*	*SD*	*M*	*SD*	*M*	*SD*
NA Inertia	0.284	0.108	0.244	0.075	0.239	0.104
PA Inertia	0.313	0.100	0.292	0.077	0.274	0.068
NA Variability	0.178	0.179	0.149	0.161	0.137	0.167
PA Variability	0.342	0.196	0.307	0.159	0.281	0.171
Bipolarity	−0.400	0.356	−0.473	0.292	−0.503	0.272
NA Level	1.470	0.370	1.382	0.274	1.378	0.264
PA Level	2.565	0.562	2.671	0.555	2.633	0.553
Drinking Level	0.837	0.732	0.760	0.626	0.607	0.906
DTC Motivation	0.241	0.316	0.115	0.184	0.088	0.163
DTE Motivation	1.232	0.489	0.870	0.462	0.795	0.492
Stress Level	2.810	0.882	2.189	0.749	2.073	0.748

*Note*. NA = negative affect, PA = positive affect. DTC = drinking to cope, DTE = drinking to enhance.

**Table 2:** Temporal stability for affect dynamic measures and associations with stress and affect.

*Wave*		*2*	*3*	*Stress*	*NA Level*	*PA Level*
NA Inertia	*1*	0.055	−0.023	0.177 *	0.174 *	0.016
	*2*		0.078 *	0.302 *	0.249 *	−0.079 *
	*3*			0.310 *	0.212 *	−0.125 *
PA Inertia	*1*	0.059 *	0.031	−0.074 *	−0.107 *	−0.092 *
	*2*		0.017	−0.002	−0.041	−0.059 *
	*3*			0.031	−0.044	−0.047
NA Variability ^a^	*1*	0.361 *	0.283 *	0.508 *	0.637 *	−0.069 *
	*2*		0.393 *	0.522 *	0.588 *	−0.162 *
	*3*			0.489 *	0.540 *	−0.205 *
PA Variability ^a^	*1*	0.315 *	0.261 *	0.081 *	0.076 *	0.130 *
	*2*		0.297 *	0.059	0.098 *	0.107 *
	*3*			−0.031	0.069 *	0.091 *
Bipolarity	*1*	0.223 *	0.183 *	−0.203 *	0.034	−0.173 *
	*2*		0.348 *	−0.137 *	0.017	−0.103 *
	*3*			−0.124 *	0.098 *	−0.076 *
NA Level	*1*	0.436 *	0.310 *	0.589 *		0.001
	*2*		0.381 *	0.626 *		−0.148 *
	*3*			0.663 *		−0.231 *
PA Level	*1*	0.469 *	0.361 *	−0.116 *		
	*2*		0.428 *	−306 *		
	*3*			−0.372 *		

*Note*: NA = negative affect, PA = positive affect. ^a^ log-transformed values. * *p* < 0.05.

**Table 3:** Models predicting mean daily drinking-related outcomes.

	*Wave 1*							*Wave 2*						*Wave 3*					
		*Model 1*			*Model 2*			*Model 1*			*Model 2*			*Model 1*			*Model 2*		
*Average Drinks*		β	SE *	*p* ^	Β	SE *	*p* ^	β	SE *	*p* ^	β	SE *	*p* ^	β	SE *	*p* ^	β	SE *	*p* ^
	NA inertia	0.036	0.047	0.235	0.048	0.044	0.141	−0.032	0.049	0.260	−0.022	0.047	0.313	0.094	0.046	0.028	0.090	0.059	0.028
	PA inertia	−0.059	0.041	0.078	−0.071	0.044	0.051	−0.011	0.051	0.427	0.002	0.050	0.483	−0.017	0.049	0.373	−0.010	0.056	0.427
	NA variability	−0.023	0.031	0.242	−0.022	0.033	0.250	−0.014	0.033	0.343	−0.018	0.036	0.318	0.043	0.040	0.142	−0.003	0.106	0.480
	PA variability	0.031	0.034	0.172	0.030	0.034	0.197	0.044	0.036	0.095	0.047	0.035	0.087	0.015	0.041	0.355	0.023	0.045	0.306
	Bipolarity	0.173	0.033	<0.001	0.165	0.033	<0.001	0.059	0.031	0.023	0.051	0.034	0.065	0.204	0.040	<0.001	0.193	0.042	<0.001
	NA level				−0.036	0.043	0.201				−0.020	0.044	0.327				0.054	0.148	0.243
	PA level				−0.042	0.042	0.157				−0.070	0.042	0.046				−0.032	0.038	0.205
*Coping motives*																			
	NA inertia	0.149	0.037	<0.001	0.028	0.034	0.208	0.127	0.054	0.004	−0.037	0.035	0.144	0.175	0.058	0.003	0.067	0.034	0.026
	PA inertia	−0.057	0.037	0.054	−0.024	0.033	0.225	−0.070	0.047	0.063	0.001	0.039	0.349	0.024	0.064	0.354	0.001	0.046	0.488
	NA variability	0.379	0.024	<0.001	−0.032	0.033	0.175	0.386	0.031	<0.001	−0.204	0.045	<0.001	0.309	0.038	<0.001	−0.249	0.039	<0.001
	PA variability	−0.142	0.028	<0.001	−0.020	0.027	0.220	−0.084	0.032	0.004	0.038	0.029	0.066	−0.102	0.037	0.007	0.038	0.032	0.118
	Bipolarity	0.077	0.028	0.002	0.006	0.025	0.396	0.151	0.033	<0.001	−0.017	0.027	0.011	0.012	0.038	0.373	−0.017	0.031	0.289
	NA level				0.579	0.025	<0.001				0.739	0.029	<0.001				0.705	0.026	<0.001
	PA level				−0.092	0.022	<0.001				−0.058	0.024	0.007				−0.058	0.029	0.102
*Enhance motives*																			
	NA inertia	0.037	0.039	0.179	0.042	0.039	0.145	0.014	0.048	0.381	0.020	0.049	0.333	−0.019	0.046	0.354	0.000	0.048	0.496
	PA inertia	−0.036	0.039	0.187	−0.032	0.039	0.198	−0.069	0.049	0.087	−0.049	0.044	0.119	−0.032	0.054	0.282	−0.004	0.051	0.469
	NA variability	0.007	0.029	0.412	0.035	0.039	0.189	0.047	0.035	0.099	0.053	0.049	0.135	−0.012	0.038	0.386	0.056	0.054	0.151
	PA variability	0.018	0.031	0.274	−0.001	0.032	0.493	0.054	0.035	0.061	0.032	0.036	0.192	0.132	0.036	<0.001	0.104	0.039	0.003
	Bipolarity	−0.172	0.029	<0.001	−0.154	0.029	<0.001	−0.081	0.035	0.011	−0.067	0.029	0.020	−0.133	0.040	0.001	−0.104	0.037	0.005
	NA level				−0.012	0.036	0.367				0.051	0.045	0.119				0.001	0.051	0.495
	PA level				0.113	0.027	<0.001				0.182	0.027	<0.001				0.186	0.034	<0.001

*Note*. Model 1: dynamic measures entered simultaneously in the same model. Model 2: dynamic measures and mean affect levels entered simultaneously in the same model. β = standardized regression coefficient. * SE is the Bayesian posterior SD. ^ One-sided *p*-value.

## Data Availability

Data is available on request from the authors.

## Supplementary Materials

The following supporting information can be downloaded at https://www.mdpi.com/article/10.3390/bs15080998/s1. Table S1: Correlations among dynamic measures within waves.
