# Supplementary material for: A Longitudinal Examination of Stress, Affect Dynamics, and Alcohol-Related Outcomes Across Emerging Adulthood
Source: Behav Sci (Basel). 2025 Jul 22;15(8):998. doi: 10.3390/bs15080998 (PMC12382984; doi:10.3390/bs15080998)
Supplement: Supplementary file 1 [file behavsci-15-00998-s001.zip › behavsci-3725045-supplementary.pdf]

**Supplemental table S1. Correlations for dynamic measures within waves**

| | | *Wave* | *2* | *3* | *4* | *5* |
|---|---|---|---|---|---|---|
| *1* | NA Inertia | *1* | .108* | .081* | -.054 | -.030 |
| | | *2* | .079* | .180* | -.031 | -.051 |
| | | *3* | .109* | .214* | -.024 | .027 |
| *2* | PA Inertia | *1* | | -.104* | -.100* | .063* |
| | | *2* | | -.030 | -.060* | .049 |
| | | *3* | | -.050 | .018 | .039 |
| *3* | NA Variance | *1* | | | .416* | -.162* |
| | | *2* | | | .326* | -.150* |
| | | *3* | | | .267* | -.208 |
| *4* | PA Variance | *1* | | | | -.137* |
| | | *2* | | | | -.134* |
| | | *3* | | | | -.207* |
| *5* | Bipolarity | *1* | | | | |
| | | *2* | | | | |
| | | *3* | | | | |

*Note***.** NA = negative affect, PA = Positive affect. *$p$<.05
